# Correlation between Interleukin-17 gene polymorphism and osteoarthritis susceptibility in Han Chinese population

**DOI:** 10.1186/s12881-018-0736-0

**Published:** 2019-01-18

**Authors:** Yuming Bai, Shijun Gao, Ying Liu, Shengli Jin, Haisen Zhang, Ke Su

**Affiliations:** 1 Department of Joint Surgery, the Third Hospital of Hebei Medical University, Shijazhuang, 050051 China; 2 Second Department of Orthopaedic, Central Hospital of Cangzhou City, Cangzhou, 061001 Hebei China; 3 Operating Theatre, People’s Hospital of Cangzhou City, Cangzhou, 061001 Hebei China

**Keywords:** IL-17F, IL-17A, Inflammation, Polymorphism, Osteoarthritis

## Abstract

**Background:**

Interleukin-17 (IL-17), a pleiotropic cytokine, plays a significant role in the inflammatory diseases. By a pilot study with small population, IL-17 polymorphisms (IL-17A rs2275913 and IL-17F rs763780) showed a more potential risk factor in knee osteoarthritis (OA) in our recruited subjects. In the current study, the association between IL-17A rs2275913 and IL-17F rs763780and the risk of OA in a Chinese population is studied.

**Methods:**

The IL-17A rs2275913 and IL-17F rs763780 polymorphisms were determined in 594 knee OA cases and 576 healthy controls, using polymerase chain reaction-restriction fragment length polymorphism assay. The relationship between genotype distribution and disease risk, as well as OA severity was analyzed by Chi-square test and multivariate logistic regression.

**Results:**

The experimental results indicated that the polymorphism in IL-17 gene rs2275913 site were related to knee OA risk after the adjustment of BMI, sex, age, smoking and drinking status (AA vs. GG: odds ratio (OR), 1.411; 95% confidence interval (CI), 1.021–1.950; *P* = 0.040; A allele vs. G allele: OR, 1.192; *P* = 0.037; 95% CI, 1.012–1.404;). Similarly, subjects who are bearing the rs763780 variant genotypes (TC and CC) and C allele also had a higher susceptibility to knee OA compared with those who are bearing the TT genotype (TC vs. TT, OR: 1.312; *P* = 0.039; 95% CI: 1.017–1.692; CC vs. TT, OR: 2.812, *P* = 0.006, 95% CI: 1.338–5.909; C allele Vs. T allele, OR:1.413, *P* = 0.002, 95% CI:1.141–1.751). In the meantime, one high-risk haplotypes, AC (OR was 7.22, *P* < 0.01) was found. Both two polymorphisms do not correlated with OA severity based on Kellgren-Lawrence (K&L) scales. Finally, serum IL-17 levels of knee OA patients were greatly higher than those of controls (*P* = 0.001).

**Conclusions:**

With the limited size sample, our study shows that IL-17 gene polymorphisms possibly related to the high-risk knee OA occurrence.

## Background

Osteoarthritis (OA), is featured by diverse changes and cartilage breakdown in the subchondral bone, resulting in chronic pain, deformity, joint swelling, and whole joint abnormalities. However, the exact cause of OA remains unknown. According to previous studies, OA has a complicated pathogenesis due to the interaction with complex pathogenic factors, including mechanical, biochemical, genetic, endocrine, metabolic, and environmental factors [1–5]. Moreover, the bony outgrowths (osteophytes), inflammation of the synovial membrane, and its triggering catabolic and pro-inflammatory mediators such as prostaglandin E2, neuropeptides, cytokines, and nitric oxide are essential to OA formation [5–7]. It is estimated that 18% of females and 9.6% of males older than 60 years suffer from symptomatic OA worldwide [8–11].

Although OA is considered a non-inflammatory condition earlier, with in-depth research on its physiopathology, now it is widely accepted that synovial inflammation is a feature of OA, and characterized by immune cell infiltration and cytokine secretion [12]. Both Innate immune system activation [13] and T-cell mediated inflammatory pathway play central roles in disease development and progression [14]. IL-17 is essential to both the adaptive and innate immune systems. It has five confirmed receptors (IL-17RA-RD and SEF) and six members (IL-17A-F) [15, 16]. IL-17A and IL-17F are secreted by Th17 cells, which is a distinct lineage of CD4+ effector cells [17]. Moreover, IL-17, as a pro-inflammatory cytokine, can trigger the release of chemokines, cytokines, antimicrobial peptides, and matrix metalloproteinases from mesenchymal and myeloid cells [18]. Previous studies also demonstrate that IL-17 is expressed in synovial tissues, and could contribute to cartilage breakdown and synovial infiltration in OA by inducing the release of chemokines by chondrocytes [19] and augments nitric oxide production in OA cartilage via nuclear factor kappa B activation [20].

Studies have reported that genetic polymorphisms of IL-17 are related to the susceptibility of a scope of inflammation-related diseases including ulcerative colitis, gastric cancer, breast cancer, and rheumatoid arthritis [21–24]. Although there is no relative report about association of IL-17 polymorphism with OA in the publicly available Genome wide association study database, we still hypothesize that IL-17 is a potential risk factor for OA pathogenesis. The correlation between the polymorphisms of IL-17A and IL-17F and the risk of knee osteoarthritis among the Chinese population has not been reported. By a pilot study with small population, IL-17A rs2275913 and IL-17F rs763780 shows more potential risk factor in OA in our recruited subjects. The present study focusses on these two SNPs to investigate their relationship with OA morbidity and severity.

## Methods

### Subjects

With all criteria adhered to the Declaration of Helsinki, this study was permitted by the Ethics and Research Committee of the Central Hospital of Cangzhou City, China. All participants provided written informed consent with gender, age, weight and body mass index (BMI) included. All participants were older than 40 years. Out of the 594 patients diagnosed with primary knee OA, 576 age-matched healthy controls free from symptoms or signs of OA. Other types of arthritis or joint diseases were enrolled in this study.

The criteria of the American College of Rheumatology was utilized in diagnosing knee OA [25], including primary OA with any symptom and radiographic sign of OA as per the Kellgren-Lawrence (K&L) scale (≥2 scale). Two independent examiners blind to the clinical data carried out the clinical examination and radiological assessment. The sex and age matched control subjects were free from personal or family history of OA. Other etiologies that cause knee diseases, such as inflammatory arthritis, post septic arthritis, and posttraumatic, developmental or skeletal dysplasia were not included.

### Sample collection

Peripheral blood was collected by venipuncture from participants into EDTA tube for DNA isolation. In addition, the biochemical parameters of the serum were detected.

### Genomic DNA isolation

DNA for genomic studies was extracted with commercial DNA Blood Mini Kit (QIAGEN, Hilden, Germany) from 200 μL of whole blood following the manufacturer’s instructions. DNA concentration was measured by spectrophotometry (NanoDrop 2000, Thermo Scientific) diluted to nearly 40 ng/μL.

### DNA sequencing analysis

IL-17 copies in the samples were measured by PCR through amplification. The primers of SNP rs2275913 were as follows: forward 5’-ATTTCTGCCCTTCCCATTTT-3′ and reverse 5′- CCAGGAGTCATCGTTGTTT-3′. For SNP rs763780, the primers used were: forward 5’-GCAGAGCACTGGGTAAGGAG-3′ and reverse 5’-CTGCATCAATGCTCAAGGAA-3′. Sequencing was carried out by a Bio-Informatic company (Life technology, Shang Hai, China).

### Serum IL-17A and IL-17F levels

Serum samples were collected from all patients and healthy subjects. The blood was allowed to clot for 30 min at 4 °C before centrifugation at 3000 rpm for 10 min at 4 °C. Total serum was separated and stored at − 20 °C until they were ready to be used. Sandwich ELISA was used to measure the concentrations of serum IL-17A and F following the manufacturer’s instructions. The intra-assay coefficients of variation came was set as 10%.

### Statistical analysis

The demographic and clinical data were shown as Mean ± SD and compared among groups by the Student’s *t*-test. The genotype and allelic frequencies were assessed by Hardy-Weinberg equilibrium (HWE) and compared by Chi-square test and Fisher’s exact test by the online calculator tool (http://www.oege.org/software/hardy-weinberg.html). Association between the SNP and the OA risk was evaluated by calculating the Odds ratio (OR) and 95% confidence interval (CI) for the additive model, the dominant model, the recessive model by using SPSS 19.0 software (IBM SPSS, USA). *P* values < 0.05 were regarded as statistically significant.

## Results

### Features of two groups are consistent

The characteristics of the two groups of 594 knee OA patients and 576 control subjects were summarized in Table 1. The mean ages of the control group and knee OA group were 58.3 ± 9.6 and 59.5 ± 8.9 years respectively. There was no significant difference in mean age, sex, drinking and smoking status between these two groups, indicating subjective matching. The mean BMI of the OA group (26.2 ± 3.8) was greatly higher compared with that of the control group (24.6 ± 3.1) (*P* = 0.0478), which is consistent with previous findings that reported high BMI in OA [26, 27].

**Table 1:** Demographic characteristics of the study population

Variables	Healthy control (*n* = 576)	Knee osteoarthritis patients (*n* = 594)	*P* value
Age (mean ± SD)	58.3 ± 9.6	59.5 ± 8.9	0.214
Gender			0.517
Male	174	201	
Female	402	393	
Body mass index (kg/m2)	24.6 ± 3.1	26.2 ± 3.8	0.0478
Smoking			
Yes	183	213	0.155
No	393	381	
Drinking			0.790
Yes	69	75	
No	507	519	
IL-17A concentration (x ± S, pg/mL)	2.98 ± 0.14	3.91 ± 0.27	0.0325
IL-17F concentration (x ± S, pg/mL)	104.5 ± 12.6	142.8 ± 14.9	0.0249

### A allele of rs2275913 and C allele of rs763780 increase the risk of knee OA

The distribution of each allele and genotype is shown in Table 2. Both SNPs were within the Hardy-Weinberg equilibrium. In terms of the genotype and allele frequencies in rs2275913 polymorphism, there was a remarkably difference between OA patients and healthy controls, which was shown in Table 2. In addition, all subjects carrying AA genotype have significantly higher risks of OA compared with GG genotype (*P* < 0.05). The results revealed that subjects with the A allele were more likely to get knee OA compared with those bearing the G allele (OR, 1.1192; 95% CI, 1.012–1.404; *p* = 0.037). Further analysis demonstrated the higher risk of AA genotype and A allele mainly existed in female sub-population (P < 0.05), but not in male population (*P* > 0.05, Table 2).

**Table 2:** Distributions of IL-17 SNPs genotypes in each group and analyses of associations between these polymorphisms and knee OA risk

Genotype	Overall (N)				Female (N)				Male (N)			
	control	OA	OR (95% CI)	*P*	control	OA	OR (95% CI)	*P*	control	OA	OR (95% CI)	*P*
Additive model												
rs2275913	576	594			402	393			174	201		
GG	207	189	1.00	1.000	145	122	1.00	1.00	62	67	1.00	1.000
GA	265	271	1.12 (0.864–1.452)	0.427	184	179	1.156 (0.842–1.587)	0.376	81	92	1.051 (0.666–1.659)	0.907
AA	104	134	1.411 (1.021–1.950)	0.04	73	92	1.498 (1.014–2.213)	0.048	31	42	1.254 (0.703–2.236)	0.466
Allele												
G	679	649	1.00	1.000	474	423	1.00	1.00	205	226	1.00	1.000
A	473	539	1.192 (1.012–1.404)	0.037	330	363	1.233 (1.011–1.503)	0.043	143	176	1.116 (0.835–1.493)	0.460
rs763780												
TT	411	380	1.00	1.000	287	246	1.00	1.000	124	134	1.00	1.000
TC	155	188	1.312 (1.017–1.692)	0.039	108	131	1.415 (1.042–1.923)	0.029	47	57	1.122 (0.711–1.772)	0.643
CC	10	26	2.812 (1.338–5.909)	0.006	7	16	2.667 (1.079–6.588)	0.033	3	10	3.085 (0.830–11.468)	0.093
Allele												
T	977	948	1.00	1.000	682	623	1.00	1.000	295	325	1.00	1.000
C	175	240	1.413 (1.141–1.751)	0.002	122	163	1.463 (1.129–1.894)	0.004	53	77	1.319 (0.899–1.935)	0.176
Dominant model												
rs2275913												
GG + GA	472	460	1.00	0.059	329	301	1.00	0.089	143	159	1.00	0.514
AA	104	134	1.322 (0.993–1.761)		73	92	1.378 (0.976–1.944)		31	42	1.219 (0.727–2.042)	
rs763780												
TT + TC	566	568	1.00	0.01	395	377	1.00	0.058	171	191	1.00	0.098
CC	10	26	2.591 (1.238–5.422)		7	16	2.395 (0.974–5.886)		3	10	2.984 (0.808–11.023)	
Recessive model												
rs2275913												
GG	207	189	1.00	0.139	145	122	1.00	0.154	62	67	1.00	0.664
GA + AA	369	405	1.202 (0.943–1.532)		257	271	1.253 (0.933–1.684)		112	134	1.107 (0.722–1.697)	
rs763780												
TT	411	380	1.00	0.007	287	246	1.00	0.01	124	134	1.00	0.372
TC + CC	165	214	1.403 (1.096–1.795)		115	147	1.491 (1.107–2.008)		50	67	1.240 (0.798–1.926)	

*OA* osteoarthritis, *OR* odds ratio, *CI* confidence interval

With regards to the rs763780 polymorphism, similar results also observed. There was a significant difference in the genotype and allele frequencies between the two groups (knee OA patients and control subject groups), which was shown in Table 2. In addition, C allele rather than T allele can increase the risk of developing knee OA (Table 2). Further study also demonstrated that C allele more caused higher risk than in T allele in female subpopulation (*P* = 0.004). In the male population, the CC genotype and C allele did not induce significant higher risk for OA. Dominant and recessive model also demonstrated the similar results that A allele of rs2275913 and C allele of rs763780 increase the risk of knee OA.

### IL-17 polymorphism is not related to OA severity

**Fig. 1:**
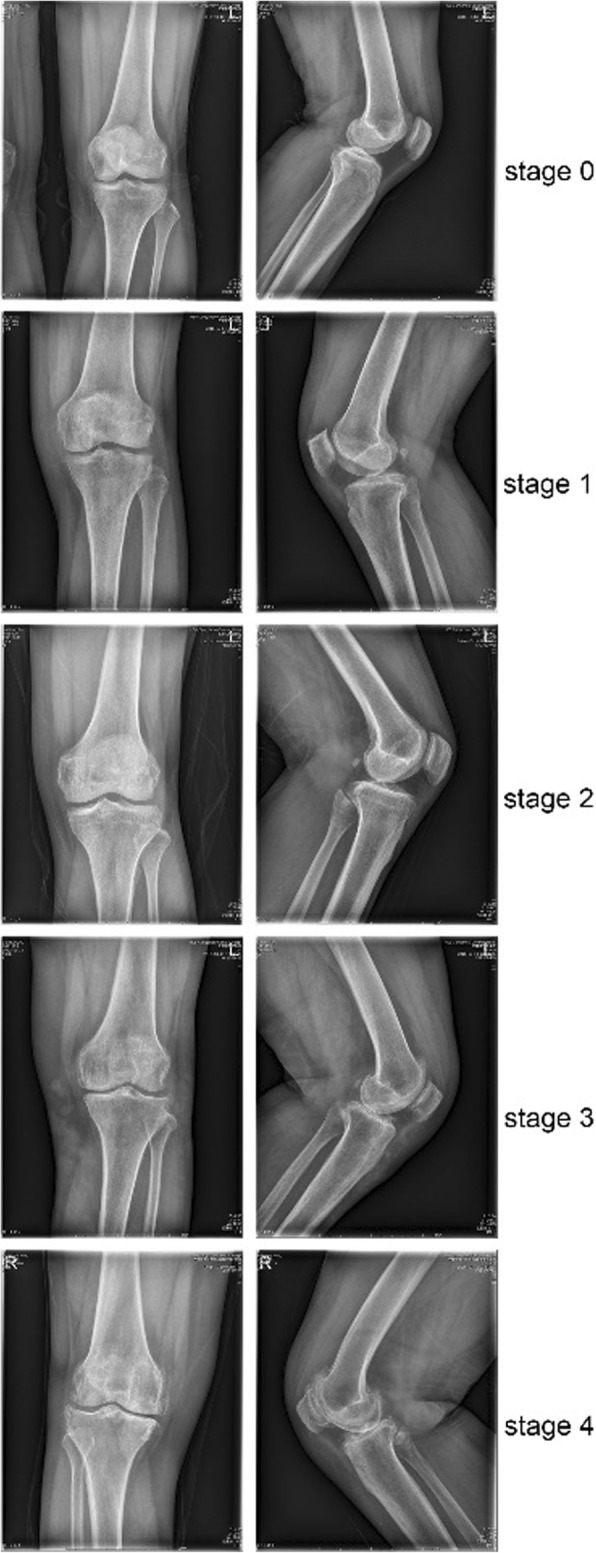
Representive photos of different severity of knee OA based on Kellgren-Lawrence (K&L) scale (from stage 0 to stage 4)

All included OA patients were over 2 Kellgren-Lawrence Grading Scales, and the typical CT-scan figures were showed per scale (Fig. 1). SNP distribution among healthy control (scale 0) and OA patients of different scale (2–4) were shown in Table 3. There is no significant difference among all the SNPs and alleles ranging from scale 2 to 4, suggesting no effect of IL-17 polymorphism on OA severity.

**Table 3:** The association of different genotypes and alleles of various polymorphisms with the severity of knee OA

Genotype	N	scale 2	scale 3	scale 4	*P* value
rs2275913	594				0.116
GG	189	63	57	69	
GA	271	78	97	96	
AA	134	29	44	61	
Allele					0.057
G	649	204	211	234	
A	539	136	185	218	
rs763780					0.272
TT	380	135	141	104	
TC	188	55	68	65	
CC	26	6	10	10	
Allele					0.077
T	948	325	350	273	
C	240	67	88	85	

### Serum IL-17A/F levels are higher in OA patients

The median serum concentration of IL-17A was 2.98 ± 0.14 pg/ mL and 3.91 ± 0.27 pg/ mL in healthy controls and OA patients, respectively (Table 1). The serum levels of IL-17A in OA patients were higher than that of healthy controls (*p* = 0.0325). The serum levels of IL-17F in OA patients were also higher than that of healthy controls (*P* = 0.0249). However, when we further summarized the average serum concentration of IL-17A and IL-17F in each genotype, there was no significant difference among all three genotypes for these two SNPs (Table 4).

**Table 4:** The serum levels of IL-17A and IL-17F in the different genotypes

Genotype	IL-17A concentration (x ± S, pg/mL)	IL-17F concentration (x ± S, pg/mL)	*P* value
rs2275913			**> 0.05**
GG	3.92 ± 0.48	142.9 ± 45.6	
GA	3.76 ± 0.35	134.5 ± 21.5	
AA	3.95 ± 0.50	143.4 ± 32.3	
rs763780			**> 0.05**
TT	3.81 ± 0.44	138.3 ± 43.4	
TC	3.75 ± 0.38	143.1 ± 52.1	
CC	3.94 ± 0.39	140.8 ± 43.8	

## Discussion

Previous studies implicated that IL-17/IL23 pathway and TH17 cells play an important role in inflammation-related diseases [28], and several meta-analysis examined the relationship between IL-17A (rs2275913) and IL-17F (rs763780) gene polymorphisms and the risk of inflammatory diseases, including periodontitis, rheumatoid arthritis (RA), and inflammatory bowel disease, and all these documents demonstrate that IL-17 polymorphisms may increased the RA risk, but dependent with race and ethnic groups [29]. Beside RA, IL17A (rs2275913) polymorphism has been reported to be associated with the development of rheumatic heart disease in south Indian population [30], and a variant of IL17F (rs763780) may contribute to the development of necrotizing enterocolitis [31]. All these publications suggest that IL17 polymorphism may widely to be associated with immune mediated diseases.

Although IL-17A and IL-17F have been reported to be associated with collagen-induced arthritis, and arthritis in experimental animal models [32], there is no study about relationship between rs2275913 (IL-17A SNP) and rs763780 (IL-17F SNP) with OA risk. Our study might be first time to investigate the involvement of IL-17 gene polymorphisms in knee OA and whether these are correlated with serum levels of IL-17. Up to date, we have yet to show whether these two SNPs affect the IL-17 secretion in the human plasma. But from data of Table 4, these two SNPs seem not influence the plasma concentration of IL-17A and IL-17F. Our results suggested that the IL-17A rs2275913 polymorphism has a significant impact on the risk of knee OA and that persons carrying the rs2275913 A allele are at a higher risk of developing knee OA as compared with those carrying the G allele. Additionally, rs763780 C allele, was also related to a greatly increased risk of developing knee OA. Further assessment of the effect of IL-17 polymorphisms on knee OA risk was stratified by sex, and showed a significant association in female patients’ subgroups.

In spite of the positive relationship between rs2275913 and rs763780 polymorphisms and the risk of knee OA observed in this study, IL-17 serum levels had no great differences in allele subgroups. These data did not show any association between IL-17 serum levels and IL-17 polymorphisms in the present study, however, a higher serum concentration was found in overall patients when it was compared with the healthy control. The limitation is that we did not test the concentration of IL-17A and IL-17F in knee joint (mainly from knee articular cavity and synovial tissues) of each patient, and local concentration of IL-17 may be more important than systemic IL-17 in plasma. Therefore, it is possible that SNPs of IL-17 might cause alteration of the IL-17 concentration in knee joint microenvironment, including knee articular cavity and synovial tissues, further influence the risk of OA development.

Patients carrying the A (rs2275913 G/A) and C (rs763780 T/C) alleles were related to increased risk for knee OA when compared to individuals who are carrying the wild-type alleles. Furthermore, we demonstrated that the correlation between the two polymorphisms under study and knee OA risk appeared significant among the female subjects; however, this evidence is just an association but not cause and effect. There is a possibility that this finding is as a result of the larger quantity of female subjects (*n* = 393) compared to the male subjects (*n* = 201), which may lead to a limited statistical power and robustness. This might also be attributed to other important factors related to women such as estrogen-relate effects and the fact that women are less exposed to tobacco smoking and heavy alcohol drinking [33, 34]. However, a larger sample size is needed to further investigate the underlying mechanisms of this association.

Aside the small sample size, we acknowledge other limitations such as the study population. With the study population confined to the population of He Bei province, the findings may not apply to other population. It would of great significance if the role of IL-17 polymorphisms in patient susceptibility to knee OA from other ethnic population is studied. Also, the present research investigated only two SNPs in the IL-17 gene. It would be of more interest to define more SNPs and investigate their role in knee OA. More importantly, population stratification—allele frequency differences between cases and controls due to systematic ancestry differences—can cause spurious associations in disease studies, and possible false positive results [35]. Therefore, more detailed statistical analysis is needed to do to make results more accurately.

## Conclusions

In conclusion, we have successfully demonstrated that functional polymorphisms of IL-17 are significantly related to the risk of knee OA. Also, the variant alleles rs2275913 AA and rs763780 CC were related to the increased risk of knee OA but not the wild-type alleles. Serum IL-17 levels significantly correlated with increased risk of knee OA which taking together might facilitate defining high risk subjects to prevent the initial development of knee OA.

## Abbreviations

BMI: Body mass index
CI: Confidence interval
HWE: Hardy-Weinberg equilibrium
IL-17: Interleukin-17
K&L: Kellgren-Lawrence
OA: Osteoarthritis
OR: Odds ratio
